# Evaluation of tuberculosis infection in COVID-19 patients: a case of tuberculosis and COVID-19 co-infection

**DOI:** 10.1186/s43162-022-00134-8

**Published:** 2022-06-02

**Authors:** Hamid Reza Niazkar, Behdad Zibaee, Seyed Behzad Razavi, Kasra Ghanaeian, Vahid Talebzadeh, Negin Haji Vosugh

**Affiliations:** 1 Breast Diseases Research Center, Shiraz University of Medical Sciences, Shiraz, Iran; 2 Student Research Committee, Gonabad University of Medical Sciences, Gonabad, Iran; 3 Internal Medicine Department, Gonabad University of Medical Sciences, Gonabad, Iran; 4 Emergency Medicine Department, Gonabad University of Medical Sciences, Gonabad, Iran; 5 Pediatrics Medicine Department, Sabzevar University of Medical Sciences, Sabzevar, Iran

## Abstract

The COVID-19 pandemic affected millions of people worldwide, becoming a challenge of every nation. Since the COVID-19 can present wide spectrum of clinical signs and symptoms, patients with symptoms similar to that of COVID-19 may be misdiagnosed during the context of COVID-19 pandemic. In this regard, various co-infections may affect the outcome of COVID-19 patients if it lefts undiagnosed, especially during the administration of immunosuppressive drugs. Similar to COVID-19, TB affect the lungs and respiratory airways primarily. These two diseases have resembling symptoms, including dry cough, fever, and dyspnea. Due to the importance of early COVID-19 diagnosis, many other respiratory infectious diseases such as tuberculosis (TB) may be missed. Herein, a case of COVID-19 and tuberculosis co-infection is presented.

## Introduction

The severe acute respiratory syndrome coronavirus 2 (SARS-COV-2) was initially identified in Wuhan, Hubei, China, in late December of 2019 [1]. The SARS-COV-2 is responsible for a multi-systemic infectious disease named COVID-19, which manifest with wide range of respiratory, gastrointestinal, neurological, and etc. signs and symptoms [2–5]. The ongoing COVID-19 pandemic has affected many lives worldwide. Until 1 August 2020, more than 17,100,000 individuals have been infected with the SARS-COV-2, resulting in more than 668,000 death cases [3, 6]. SARS-COV-2 can easily spread through respiratory droplets and close individual contacts [7]. Infected individuals are assumed to transmit the COVID-19 during the incubation period, which is estimated to be about 2 to 14 days. While some of COVID-19 patients are known to be asymptomatic, symptomatic patients mostly present with cough and fever. Other symptoms include dyspnea, myalgia, confusion, headache, diarrhea, nausea, and vomiting [1, 2, 8, 9].

Social distancing and early isolation of infected individuals play crucial roles in the controlling the COVID-19 outbreak. Furthermore, the definite diagnosis of COVID-19 is made based on the real-time polymerase chain reaction (RT-PCR). However, physicians’ clinical suspicion is still the leading part of diagnosis. Since the clinical sign and symptoms of COVID-19 are very similar to that of various respiratory diseases, the diagnosis of COVID-19 can be challenging. In this regard, the chest computed tomography (CT) scan such as ground glass opacity and hematological findings (lymphocytopenia) can be helpful [1]. Nevertheless, due to the context of COVID-19 pandemic, and its importance, many other respiratory infectious diseases such as tuberculosis (TB) may miss [10]. In this regard, TB is the cause of more than 1.2 million deaths per year, globally. COVID-19 and TB are diseases that affect the lungs and respiratory airways primarily. These two diseases have resembling symptoms, including dry cough, fever, and dyspnea [11]. Herein, a case of COVID-19 and tuberculosis co-infection is presented.

## Case report

A 96-year-old woman was admitted to our hospital complaining of cough, fever, and dyspnea which was started from 5 days ago, and it got worse within last 3 days. She was a known case of hypertension and dyslipidemia. Physical examination revealed an axillary temperature of 38 centigrade and a diffuse crackle in lung auscultation. Ear, nose, and throat examinations were normal, and no exudates or erythema were observed. Laboratory investigations indicated a lymphocytopenia, with the absolute count of 900 (30%) lymphocytes, and 1900 (65%) neutrophils. The erythrocyte sedimentation rate (ESR) and C-reactive protein (CRP) were also elevated and reported to be 119 and + 3, respectively. Arterial blood gas (ABG) assessment were normal (PH: 7.38, PCO2: 45, and HCO3: 26), and the SpO_2_ level were 84%. High-resolution computed tomography (HRCT) of lungs revealed typical characteristics of COVID-19 and a suspected TB cavity. Furthermore, a purified protein derivative (PPD) test was conducted, and after 72 h, the induration around the injection point was 25 mm. Then, sputum smear and culture study were performed for the patient, and they were both positive for tuberculosis. Also, the COVID-19 RT-PCR test was found to be positive.

Patient treatment was started with CPAP:5 and FiO_2_: 40%, and the SpO_2_ was increased to 93%. The hydroxychloroquine was begun for the patient twice a day with the first day dose of 800 mg and then 400 mg per day. Also, 1 g of ceftazidime was given to the patient three times a day. The atorvastatin and the aspirin ordered daily, 20 mg, and 80 mg respectively. Tuberculosis treatment was also added for the patient after the diagnosis (daily dose of isoniazid 300 mg, rifampicin 600 mg, ethambutol 1200 mg, and pyrazinamide 1500 mg for four month, followed by 2 months of isoniazid 300 mg, and rifampicin 600 mg). After 10 days, the patient’s general condition got better. White blood cell (WBC) and lymphocyte count increased relatively to 4700 and 1600. The patient was free of dyspnea, fever, and a mild cough, and the SpO_2_ rate was 94% without any additional help. After discontinuing hydroxychloroquine, the patient was released to be home for further quarantine. During the follow-up for 3 weeks, the patient was completely free of any signs and symptoms.

## Discussion

The administration of Bacillus Calmette-Guérin or BCG vaccine is assumed to decrease the COVID-19 spreading and improve host response against COVID-19 infection probably through heterologous effects of adaptive immunity and trained innate immunity [12]. However, further studies are required to support this hypothesis. According to the studies, the presence of tuberculosis can be a risk factor for the rapid progression of COVID-19. Moreover, individuals with active TB tend to manifest more severe signs and symptoms [13]. Nevertheless, since these two respiratory infections present with similar signs and symptoms, the co-infection of TB can be easily missed in the context of COVID-19 pandemic. Also, physicians should be aware of possible latent TB in susceptible patients, since the TB tends to progress over a longer time interval and can remain silent after initial infection. In our patient, despite various comorbidities such as old age, hypertension, atherosclerosis, and dyslipidemia, the timely diagnose and management of pre-existing tuberculosis inevitably affected the patient’s outcome.

**Fig. 1:**
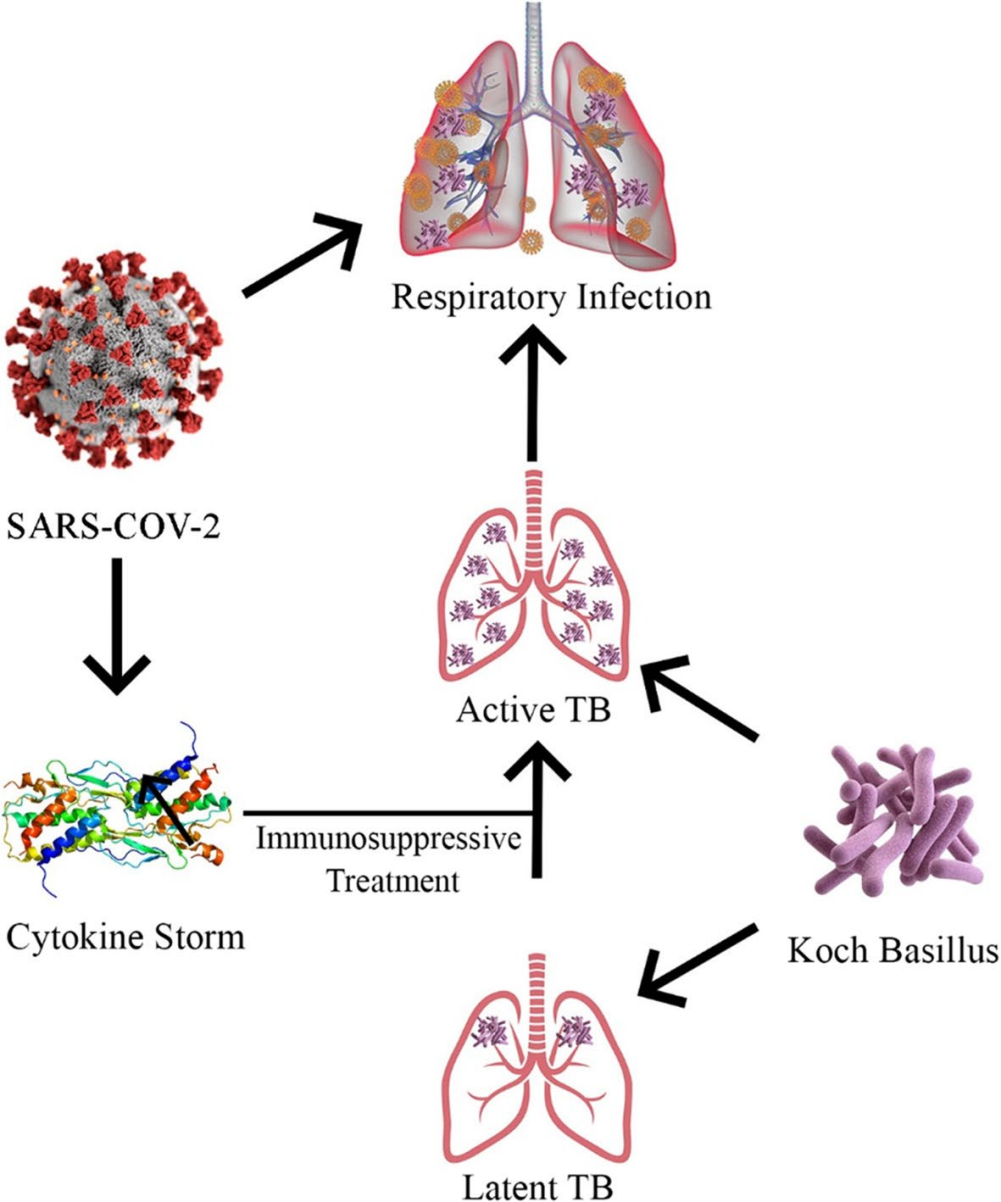
SARS-COV-2 and TB co-infection

Studies have shown that a pre-existing upper respiratory tract viral infection may susceptible the patients to a severe bacterial pneumonia. A study by Johansson et al. demonstrated that patients with both viral and bacterial pneumonia presents more severe symptoms [14]. In this regard, similar to other viruses, SARS-COV-2 may also prone the patient to a severe bacterial pneumonia. Therefore, further evaluation of pre-existing respiratory infections such as TB in COVID-19 patients of endemic areas are suggested. Also, the administration of immunosuppressive drugs such as corticosteroids in such cases could inevitably flare the possible latent TB infection (Fig. 1).

Overall, considering the inevitable negative consequences of undiagnosed TB in COVID-19 patients, the authors suggest the following criteria for TB testing in COVID-19 patients, named as “ZIBAKAR” criteria (Table 1):

1. COVID-19 patients in areas with a high prevalence of TB such as, India, China, Indonesia, Philippines, Pakistan, Nigeria, Bangladesh, South Africa, and etc.
2. COVID-19 patients with a travel history to TB endemic areas
3. COVID-19 patients with high risk for TB transmission, such as HIV positive patients, homeless individuals, IV drug abusers, and immunocompromised patients
4. COVID-19 patients with recent family history of TB or close contacts with known cases of TB.
5. COVID-19 patients with either exclusive TB radiological findings such as cavity, fibronodular opacities in the apical and upper lung zones, and etc., or atypical radiological findings of COVID-19
6. COVID-19 patients with typical TB extrapulmonary manifestations such as chronic lymphadenopathy, exudative pleural effusion, joint inflammation, sterile pyuria, pericardial effusion, etc.
7. COVID-19 patients with unexplained atypical laboratory findings such as an extreme increase of ESR, which can be justified with TB diagnosis

**Table 1:** Suggested criteria for TB testing in COVID-19 patients, named as “ZIBAKAR” criteria

1	COVID-19 patients in areas with a high prevalence of TB
2	COVID-19 patients with a travel history to TB endemic areas
3	COVID-19 patients with high risk for TB transmission
4	COVID-19 patients with recent family history of TB or close contacts with TB cases
5	COVID-19 patients with either exclusive TB radiological findings or atypical radiological findings of COVID-19
6	COVID-19 patients with typical TB extrapulmonary manifestations
7	COVID-19 patients with unexplained atypical laboratory findings which can be justified with TB diagnosis

## Conclusion

During the ongoing COVID-19 pandemic, further evaluation of the pre-existing respiratory infections may improve the prognosis of COVID-19 patients. In this regard, evaluation of tuberculosis infection in susceptible individuals and in those with one of the “ZIBAKAR” criteria are suggested. Moreover, further studies are suggested in a bid to shed the light on the correlation of COVID-19 and tuberculosis.

## Data Availability

Not applicable
